# Environmental Influences Measured by Epigenetic Clock and Vulnerability Components at Birth Impact Clinical ASD Heterogeneity

**DOI:** 10.3390/genes12091433

**Published:** 2021-09-17

**Authors:** Viviane Neri de Souza Reis, Ana Carolina Tahira, Vinícius Daguano Gastaldi, Paula Mari, Joana Portolese, Ana Cecilia Feio dos Santos, Bianca Lisboa, Jair Mari, Sheila C. Caetano, Décio Brunoni, Daniela Bordini, Cristiane Silvestre de Paula, Ricardo Z. N. Vêncio, John Quackenbush, Helena Brentani

**Affiliations:** 1 Departamento & Instituto de Psiquiatria, Faculdade de Medicina FMUSP, Universidade de São Paulo, São Paulo 05403-903, SP, Brazil; vivineri@gmail.com (V.N.d.S.R.); tahira.ana@gmail.com (A.C.T.); vinidg@gmail.com (V.D.G.); paulacmari@gmail.com (P.M.); joanaportolese@gmail.com (J.P.); ceciliafeio@gmail.com (A.C.F.d.S.); lisboa.bianca@gmail.com (B.L.); 2 Instituto Butantan, São Paulo 05503-900, SP, Brazil; 3 Laboratório de Pesquisas Básicas em Malária—Entomologia, Seção de Parasitologia—Instituto Evandro Chagas/SVS/MS, Ananindeua 66093-020, PA, Brazil; 4 Departamento de Psiquiatria, Universidade Federal de São Paulo (UNIFESP), São Paulo 04023-062, SP, Brazil; jamari17@gmail.com (J.M.); sheilaccaetano@gmail.com (S.C.C.); danibordini4@gmail.com (D.B.); csilvestrep09@gmail.com (C.S.d.P.); 5 Centro de Ciências Biológicas e da Saúde, Universidade Presbiteriana Mackenzie (UPM), São Paulo 01302-907, SP, Brazil; debruno46@gmail.com; 6 Departamento de Computação e Matemática FFCLRP-USP, Universidade de São Paulo, Ribeirão Preto 14040-901, SP, Brazil; rvencio@usp.br; 7 Center for Cancer Computational Biology, Department of Biostatistics and Computational Biology, Dana-Farber Cancer Institute, Boston, MA 02115, USA; johnq@jimmy.harvard.edu or; 8 Department of Biostatistics, Harvard T. H. Chan School of Public Health, Boston, MA 02115, USA

**Keywords:** ASD, risk factors, vulnerability components, methylation, exome, psychiatry

## Abstract

Although Autism Spectrum Disorders (ASD) is recognized as being heavily influenced by genetic factors, the role of epigenetic and environmental factors is still being established. This study aimed to identify ASD vulnerability components based on familial history and intrauterine environmental stress exposure, explore possible vulnerability subgroups, access DNA methylation age acceleration (AA) as a proxy of stress exposure during life, and evaluate the association of ASD vulnerability components and AA to phenotypic severity measures. Principal Component Analysis (PCA) was used to search the vulnerability components from 67 mothers of autistic children. We found that PC1 had a higher correlation with psychosocial stress (maternal stress, maternal education, and social class), and PC2 had a higher correlation with biological factors (psychiatric family history and gestational complications). Comparing the methylome between above and below PC1 average subgroups we found 11,879 statistically significant differentially methylated probes (DMPs, *p* < 0.05). DMPs CpG sites were enriched in variably methylated regions (VMRs), most showing environmental and genetic influences. Hypermethylated probes presented higher rates in different regulatory regions associated with functional SNPs, indicating that the subgroups may have different affected regulatory regions and their liability to disease explained by common variations. Vulnerability components score moderated by epigenetic clock AA was associated with Vineland Total score (*p* = 0.0036, adjR^2^ = 0.31), suggesting risk factors with stress burden can influence ASD phenotype.

## 1. Introduction

Autism spectrum disorder (ASD) is a neurodevelopmental disorder characterized by alterations in social communication and the presence of stereotyped and restrictive behaviors. It is a polygenic multifactorial disorder with a complex genetic architecture and a heterogeneous clinical presentation [1,2].

The genetic component seems to have a major role in ASD vulnerability. The genetic risk derives mostly from common genetic variants with a smaller contribution from de novo and rare inherited variation (2.6% of the variance in liability) [3]. In some ASD cases, a polygenic variation additively with strong acting de novo variants could increase the risk [4]. The polygenic model assumes that many inherited variants contribute to ASD, each with a small effect. This model is supported by many aspects, such as more than ten times increased risk for autism in siblings of probands, and observation of sub-threshold autistic-like traits in ASD first-degree relatives [5]. A polygenic liability to psychiatric disorders has been discussed by studies showing genetic correlation among psychiatric disorders, suggesting that they share common genetic factors [6,7]. Accordingly, not only ASD but also other psychiatric disorders are more common among relatives of ASD cases [8,9].

The heritability of ASD is estimated to be around 50%, although varying between different studies [3,10], which shows that environmental factors are also important. Different studies have shown an association between intrauterine stress exposure and a greater risk for ASD [11,12,13]. Many environmental factors associated with ASD can change epigenetic status, such as DNA methylation, and affect regulatory mechanisms in brain development [11,12,13,14,15,16,17]. Genetic liability in combination with environmental factors crosses a risk threshold triggering the disorder development [11,18]. Recent studies showed that variability in methylation levels in some genomic locations could be better explained by a combination of genetic (G) and environmental (E) factors [19,20,21,22,23,24,25]. These regions, named variably methylated regions (VMR), are located close to other functional genomic variations and enriched with CpGs sites that could influence gene expression. These VMRs did not change with age and are over-represented in Genome-Wide Association Studies (GWAS) of both psychiatric and other complex diseases, possibly representing biological markers [19].

Besides the heterogeneous etiopathology, the ASD clinical presentation is also highly heterogeneous and its dimensional diagnosis represents the idea that its features fall along a continuum of severity [26,27,28]. It has been shown that besides core symptoms, intellectual function, language abilities, and functionality contribute to this heterogeneity and severity of clinical presentation [29]. Functionality measured by the Vineland scores seems to have greater variability than ASD symptoms and has an important impact on ASD trajectories [30]. To better understand the phenotypic heterogeneity and the severity spectrum, different studies searched for ASD subgroups based on phenotype or genetic variations, but none considered environmental influences [2,18,27,28,31,32,33].

We hypothesize that a model including environmental exposure factors in addition to genetic ones could better explain the severity spectrum. The prenatal period is probably the stage in which ASD environmental risk factors interacting with genetic play their major role, however epigenetic programming, altered by environmental factors during infancy, has an important impact on neurodevelopmental trajectories [34] and could contribute to the severity spectrum separated from gestational exposure.

Differences between epigenetic and chronological age (age acceleration, AA) have been associated with stress exposure [35,36]. This mechanism, known as ‘Epigenetic age’, has the potential to assess an individual’s biological age based on DNAm levels. This measurement can show the cumulative effect of environmental factors on the epigenetic maintenance system, being used as a proxy of postnatal stress exposures [37]. So, we used AA as a proxy of postanal environmental exposure. To study gestational environmental exposure, considering that ASD vulnerability probably arises due to a genetic and gestational exposure interaction model [11,12,13] we used a Principal Component Analysis (PCA) to capture the most important factors of vulnerability. We searched principal components based on familial psychiatric history, pregnancy complications, psychosocial (maternal schooling, social class, stress), and toxic environmental exposures (tobacco, alcohol, drugs) during gestation. The genetic component was assessed based on family history considering its association with polygenic risk scores [38] and the greater contribution of common variation in ASD risk [3]. As the objective of PC analysis was to find components of vulnerability represented by genetic and gestational exposure to better explore individuals with a distinct gradient of vulnerability we search if they have differences in methylation patterns enriched by VMRs associated with genetic and environmental factors that did not change according to age. As it would be expected to find de novo and very rare variants (MAF < 0.005) with higher effects for ASD with low IQ [4,39,40], we performed a whole-exome analysis in a subsample to check if any individuals assigned to different gradients of vulnerability according to our PC analysis was not a consequence of bearing more rare genetic variations with higher functional impact.

Our results showed two components of ASD vulnerability: one is more associated with psychosocial stress exposure and the other with a biological vulnerability. A gradient of psychosocial stress exposure was detected and split into two groups, above and below average. A biological difference along this gradient was supported by a different pattern of VMRs between formed subgroups. The principal components and age acceleration were associated with Vineland scores measured at 7 years of age. The current study suggests that environmental factors can contribute to ASD phenotypic severity spectrum.

## 2. Materials and Methods

### 2.1. Participants

The sample of the present study was obtained from a Brazilian population that lives in São Paulo city and contained 68 ASD children with blood samples available from a previously published randomized clinical trial of a video modeling parenting training [41]. One patient that did not complete the Risk Scores questionnaire used to calculate the Risk Scores was excluded from the PCA analysis. The inclusion criteria were: patients with ASD diagnosed using ADI-R, ages between 3 and 7 years old, and IQ between 50 and 75. Exclusion criteria were the presence of known genetic syndrome evaluated by a clinical geneticist and individuals without the Risk Scores evaluation.

Patients were diagnosed using Autism Diagnostic Interview-Revised (ADI-R) [42], a 93-item structured interview that is conducted with parents to measure the following: reciprocal social interactions, communication, language, and behavior patterns [43]. The revised version has been abridged and modified to suit children with a mental age of approximately 18 months to adulthood and is linked to the DSM-IV and ICD-10 criteria. The interview lasts approximately 1.5 h for children up to 4 years old and becomes a little longer for older children. The score is based on the interviewer’s judgment regarding the codes that best represent the behaviors described by the respondent, where the cutoff points for the diagnosis are 10 points for the total, 10 points for item B (Social Interaction), 7 or 8 points for item C (Communication, 8 for verbal patients and 7 for non-verbal patients) and 3 points for item D (restricted and repetitive behavior patterns).

IQ assessed by the Snijders–Oomen Nonverbal Intelligent Test—Revised (SON-R 2½–7) [44] used in children of 2 years and a half to 7 years. It evaluates the cognitive skills without using verbal or written language. ASD patients’ functionality was assessed with Vineland Adaptative Behavior Scale [45], which measures adaptative behavior in domains related to communication, social skills, and daily life activities.

ASD severity was accessed with the Childhood Autism Rating Scale (CARS) [46,47], an instrument used to assess the severity of autism symptoms. The scale assesses the patient’s behavior through an informant (parents or guardians) in 15 domains that include: personal relationships, imitation, emotional response, body use, use of objects, response to changes, visual response, auditory response, response, and use of taste, smell and touch, fear or nervousness, verbal communication, non-verbal communication, activity level, level and consistency of intellectual response and general impressions of the examiner. Scores between 30 and 36 indicate mild to moderate autism and scores above 36 indicate severe autism.

The patients’ internalizing and externalizing symptoms with the Child Behavior Checklist CBCL [48]. The CBCL is part of an assessment system developed by Achenbach and Rescorla [49] that assesses children’s behavior by age group. There are two versions: one for children aged 1 1⁄2 to 5 years and one for children and teenagers aged 6 to 18 years. In both instruments, the informant should be the parents or caregivers. The first version is composed of 99 sentences that must be evaluated by the respondent as not true—as far as is known; a little true or sometimes true; or very true or often true, which corresponds respectively to 0, 1, and 2 points on the scale. The version for children and adolescents aged 6 to 18 years comprises 138 sentences, of which 118 refer to behavior problems and 20 to social competence. This scale consists of 20 items that include the child’s activities, such as games, games, and tasks; participation in groups; relationship with family and friends; independence to play and school performance. In most of these items, parents are asked to compare their children’s behavior with that of other children of the same age, in terms of performance and time spent on each activity, marking it as below average, within average, or above average. The instrument assesses emotional reactivity, anxiety/depression, somatic complaints, attention problems, aggressive behavior, sleep problems, social problems, thinking problems, and rule violations. From the scores obtained on these scales, the child or adolescent can be included in the clinical, borderline, or normal ranges, in relation to their global functioning and internalizing and externalizing profiles. For inclusion in the internalizing profile, the items are considered isolation, somatic complaints, anxiety/depression, and for inclusion in the externalizing profile, the items evaluated are violations of rules and aggressive behavior. Carvalho et al., [50] report that the CBCL is indicated in the literature as one of the most effective instruments in quantifying parental responses about their children’s behavior.

The final sample used contains n = 67 children and mother pairs: 55 males (82.1%), ages between 3 and 7 years old (mean 4.7, SD 1.3). IQ between 49–75 (mean 59.0, SD 8.8), CARS between 24–52 (mean 36.86, SD 6.39), CBCL Internalizing Symptoms between 3–21 (mean 10.56, SD 4.55), CBCL Externalizing Symptoms 3–34 (mean 13.26, SD 6.85) and

ADI-R scores between 25–60 (mean 47.0, SD 7.0). The clinical genetic examination was evaluated by a team of geneticists and no genetic syndromes were described.

Blood and data were collected at baseline for 66 children for methylation analysis and an arbitrary subsample of 33 children, mother and father trios for exome analysis. The present study was approved by the Hospital das Clinicas (University of São Paulo Medical School) Ethical Committee (CAAE: 57067016.2.0000.0068, assessment #1.637.312). All experiments were performed following the ethical principles for medical research involving human subjects (Declaration of Helsinki), Informed consent was signed by the legal guardians of all patients.

### 2.2. Risk Factor Variables

The risk factor was scored according to (a) Family social class [51,52] measured by Criterio Brasil [53], (b) Maternal schooling [52], (c) Maternal stress during gestation (familial fights, illness, or death in the family, verbal or physical aggression, and emotional or depression symptoms during pregnancy) [14,54], (d) Toxic environmental exposures (Tobacco, alcohol and drug, exposure to pesticides, and use of known deleterious medications during pregnancy), (e) Familial psychiatric history (ASD, schizophrenia, depression, bipolar disorder, alcohol and/or drug abuse, obsessive-compulsive disorder, anxiety, attention deficit, and hyperactivity disorder) [18] and (f) Gestational complications: Eclampsia/preeclampsia, gestational diabetes and excess of weight gain during gestation (Appendix A and Table S1).

### 2.3. Statistical Analysis

The statistical language R (v3.5.2) on RStudio was used for all statistical analyses. For descriptive analyses, mean scores, standard deviations (SD), and frequencies were calculated. To achieve comparability among variables, scores were transformed into standardized z-scores. A principal component analysis (PCA) was performed with the risk factors variables using the “prcomp” function with default parameters, except for the scale set to TRUE. Exposure factors represented in at least 30% of the respondent sample were included in PCA analysis. Only components with an eigenvalue greater than one were retained. To select variables with important contributions to PCs compositions we considered correlations >0.70 between variables and components.

### 2.4. Methylation Analysis

For the methylation analysis, DNA from whole blood samples was treated using the EZ DNA Methylation kit (Zymo Research, Irvine, CA, USA). The bisulfite-converted DNA was hybridized in the BeadChip Human Methylation 450 microarrays (Illumina Inc., San Diego, CA, USA) following the manufacturer’s protocol. Raw data was extracted by iScan SQ scanner using the GenomeStudio software (v.2011.1), with the methylation module v.1.9.0 IDAT files. Raw data is available at the GEO database (GSE164563).

Data analysis was performed using the minfi package [55] and other Bioconductor packages [56] (http://www.bioconductor.org, accessed on 15 September 2017). Quantile normalization [57] performed signal intensity normalization of Infinium I and II probes and ‘noob’ method (Normal-exponential convolution using out-of-band probes) background correction [58]. Batch effects were corrected using the ComBat [59] from the ChAMP package (v. 2.8.3) [60]. Cell composition was estimated based on the Houseman method [61], and no difference was found between the groups. Quality control steps removed 2784 probes (detection *p*-value > 0.01), 11,214 probes in sex chromosomes, 16,474 probes in SNPs and 26,480 located in cross-reactive sites [62], resulting in 428,560 for the differential analysis.

Identification of differentially methylated probes (DMPs) was performed with M-values (logit of β-values) employing an empirical Bayesian framework linear model from the limma package [63,64], sex factor was used as an adjusted variable Only DMPs with adjusted *p*-value < 0.05, corrected for multiple comparison effects by the Benjamini and Hochberg method, were considered further. Functional Epigenetic Modules (FEM) package (v.3.10.0) was used to find the differential methylation interactive hotspots [65]. DNA methylation age and age acceleration (AA) were calculated using Horvath’s epigenetic clock [36].

### 2.5. Enrichment Analyses rVarbase and VMRs

A set of 1000 random groups was created based on CpG coordinates from the Illumina Infinium Human Methylation 450K BeadChip platform. The specific number of probes selected for each group was based on the number of CpGs analyzed for each cluster. Coordinated comparison of CpGs/random groups and rVarBase [66] database were performed to calculate overlapping elements in rVarBase using BEDTools [67] and estimate the *p*-value. For VMRs enrichment analysis, the Multivariate State Estimation Technique (MSET) algorithm [68] with 10,000 permutations was used. We used VMRs lists from 4 different studies: Islam et al. [23], Teh et al. [20], Garg et al. [25], and Hachiya et al. [19] (Databases are described in detail in Appendix B).

### 2.6. Whole-Exome Analysis

The whole-exome was performed using the SureSelectXT HumanAllExonV5+UTR Reagent Kit (Agilent Technologies Inc, Santa Clara, CA, USA) and sequenced using HiSeq 2500 (Illumina Inc., San Diego, CA, USA). Fragments were aligned to hg19 reference genome using Burrows-Wheeler Alignment (BWA) (v.0.6.1-r104) [69], samtools (v.1.6) [70], and variant calling was performed with GATK (Genome Analysis Toolkit, v. 2.8) [71].

PLINK (v. 1.9) [72] and BEDTools (v.2.25.0) [67] were used to confirm the family relationship between samples. Only reads with Phred ≥ 30 and bases with at least 20× reads in all members of the family were considered. Variant annotation was performed with ANNOVAR (22 June 2016) [73].

Rare (MAF ≤ 0.01) and novel variants were selected according to the databases:

1000 Genomes Project [74] (http://www.1000genomes.org/, accessed on 2 February 2017), NHLBI GO ESP 6500 exomes (http://evs.gs.washington.edu/EVS/, accessed on 2 February 2017) and ExAC project (http://exac.broadinstitute.org/, accessed on 2 February 2017) [75]. Only nonsynonymous de novo (homozygous loci for the reference allele in the parents) and rare variants were selected (missense, nonsense, splice-site SNVs and frameshift indels). The genes affected by the selected variants were compared to a gene list with the 25% genes most intolerant to mutation according to the RVIS scoring method [76]. Exome data is available at PRJNA525890.

### 2.7. Regression Models

All linear regression analyses were done using the normal distribution of errors in the R core base package. Dependent variables used were represented by phenotype scores measured at the baseline of the clinical trial study and the independent variables were sex, age, and vulnerability scores.

## 3. Results

### 3.1. Participants Characteristics

The presented psychosocial, genetic, and epigenetic data was obtained for the current study from a clinical sample participating in a recently published randomized clinical trial [41]. There are n = 67 mother and child pairs being considered here, engaged before the trial’s randomization phase.

The range for maternal schooling was 16% with incomplete primary education to incomplete high school, 52% with complete high school, and 32% with higher education. Most of the families were from B, C, and D social classes, with only one family from social class A (higher); 56% from class B; 29% class C, 15% class D (lower) [77]. During gestation, 37 women (54%) did not report any kind of stress; however, 27 (39%) had 1 to 3 stressful events. Medical complications (gestational diabetes, preeclampsia, and more than 12 kg gain during gestation) were present in 38 women (55%), however, most of them had only one type (28 women, 41% of the total). The toxic environmental exposure was reported in 12 women (18%), six women reported alcohol exposure, 5 women reported smoking during gestation, and 1 reported drug use, therefore we did not consider those variables to search principal components. A family history of psychiatric disorders was present in 43 families (62%). There were 7 families (10%) that reported other cases of ASD in the family. Depression and Schizophrenia were the most prevalent disorders among the families. The first was reported in 32 relatives (from 24 families), and the second in 12 (from 11 families). The paternal age at birth was assessed and 41 families (62%) presented this information with an average value of 33.42 (SD ± 7.73).

### 3.2. Finding Components of Vulnerability

Considering as important exposure factors the ones represented in more than 30% of the respondent mothers’ sample, we performed a PCA analysis. Two principal components (PCs) had eigenvalues >1, explaining up to 60% of the variance (Table 1).

**Figure 1:**
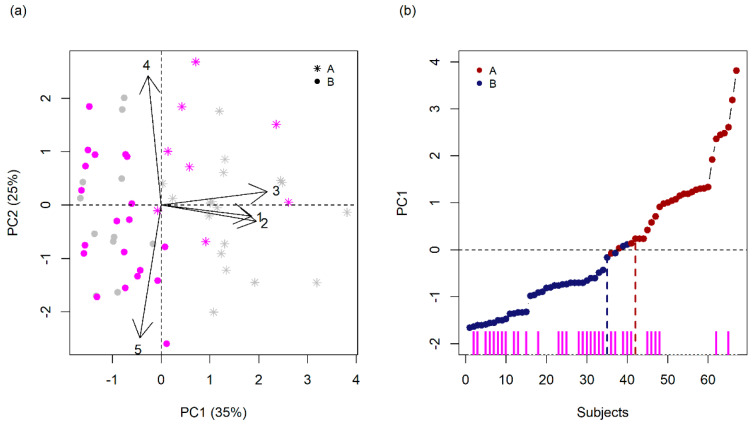
PCA identification of Psychosocial stress Vulnerability (PV) and Biological Vulnerability (BV) components. (**a**) PCA Biplot, corresponding percentage variances, and average PC1 and PC2 values (dashed lines). Original variables contributions are estimated by their eigenvectors (arrows): 1-Maternal Stress; 2-Maternal Schooling; 3-Social Class; 4-Gestational Complications; 5-Psychiatric Family History. Group A and B are represented by an asterisk and dot, respectively (**b**) Definition of above (A, red) and below (B, blue) PV average groups. Vertical lines mark A and B separation and a buffer zone in between. An arbitrary subsample had their exome examined (magenta in both panels). Whole-exome sequenced samples are represented in magenta.

PC1 has a higher correlation with Maternal Stress, Maternal Education, and Social Class, while PC2 has a higher correlation with Psychiatric Family History and Gestational Complications (bold in Table 1). In this analysis, we can also see that PC3 appears with an eigenvalue of almost 1 and it adds 18% to the first two for a cumulative variance of 78%, however, none of the variables were correlated equal or greater than 0.7 with PC3. Figure 1a shows PC1 and PC2 projection in a biplot. The first PC was interpreted as a **Psychosocial stress Vulnerability** component (PV), while the second PC was interpreted as a **Biological Vulnerability** component (BV).

We defined two groups: above (A, n = 29) and below (B, n = 38) average PC1 values (red and blue groups in Figure 1b, respectively). Aiming robustness, instead of a hard split between the groups around the average value, a buffer zone was defined, and their grouping was purposely mixed alternately (subjects between vertical lines in Figure 1b).

Statistical analysis did not show any significant differences between A and B groups regarding the clinical variables (Table S2). Evidently, the groups presented differences related to exposure to psychological and social stressors, the main contributors to PC1 (Maternal Stress, *p*-value = 3.1 × 10^−4^; Maternal Schooling, *p*-value = 2.1 × 10^−7^, and Social Class, *p*-value = 9.8 × 10^−5^). Although there was no difference regarding gestational complications, the group with higher psychosocial stress scores presented lower family history scores (*p*-value = 0.0340).

### 3.3. Methylation Analysis

Methylation analysis was performed with two different aims, the first was to search biological differences between individuals assign to different gradients of vulnerability according to our PC analysis, and the second was to assess AA as a proxy of postnatal stress exposure. To investigate if the differences observed in PV were also reflected in the children methylomes, we searched for DMPs between above (group A) and below (Group B) average PV values on PC1 (Figure 1b). After the probe exclusion described in the Methods section, 428,560 CpGs sites remained for further analysis. A total of 11,879 DMPs (corrected *p*-value < 0.05) were identified when comparing A and B groups. To verify if these DMPs could be altered in the brain, we searched on the BECon datasets [78], which presents a correspondence analysis between blood and 3 brain regions (BA7, BA10, and BA20). A total of 11,384 (96%) and identified CpGs with at least a correlation |r| ≥ 0.5 (BA7 = 384 CpGs, B10 = 416 CpGs and B20 = 362 CpGs), with a low CpGs overlap among brain regions (Figure S1), resulting in 964 (~8%) CpGs with correspondence between blood and brain.

**Figure 2:**
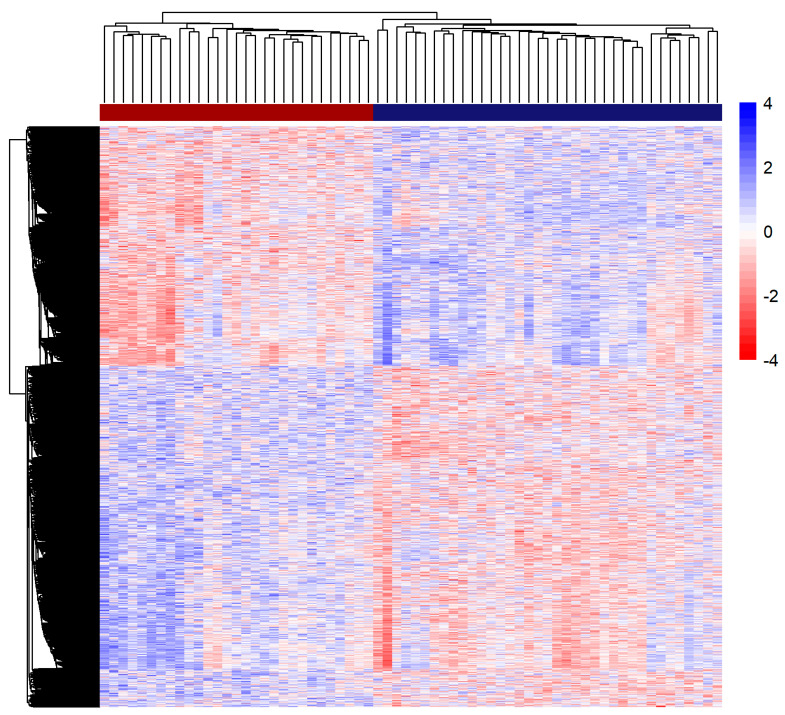
Heatmap of VMR-enriched DMPs. Each line represents the z-score normalized M-value of a CpG site (higher and lower methylation color-coded as blue and red, respectively). Clusters found in methylomics unsupervised analysis (upper bar, Group A in dark red an Group B in dark blue) coincide with above and below average groups of PC1.

Differences in children’s methylomes suggest the presence of CpG regions that vary according to differences measured in mother-based PV. To support these findings, we compared our results with public datasets, one regarding VMRs, which represent methylated regions that vary according to genetic and environmental factors. Comparison of DMPs with all VMR datasets currently openly available showed that our set is enriched for VMRs, and the majority was best explained by environmental and genetic influences (Table 2). Using the DMPs present in the VMRs database, we performed an unsupervised hierarchical clustering analysis which yielded a clear two groups pattern that recovered the original A and B partition (Figure 2).

Analyzing our DMPs with rVarbase [66], which contains regulatory regions harboring annotated SNPs, there was an overlap of 2285 CpGs, although without enrichment when compared to random groups (*p* = 1). Using these 2285 overlapping CpGs, we observed that in group B (1326 CpGs), hypermethylated CpGs were more localized in active promoters and gene enhancers, whereas in group A (959 CpGs) they were more localized in bivalent promoters and enhancers (Χ^2^-test, *p*-value < 0.05, Table S3).

**Figure 3:**
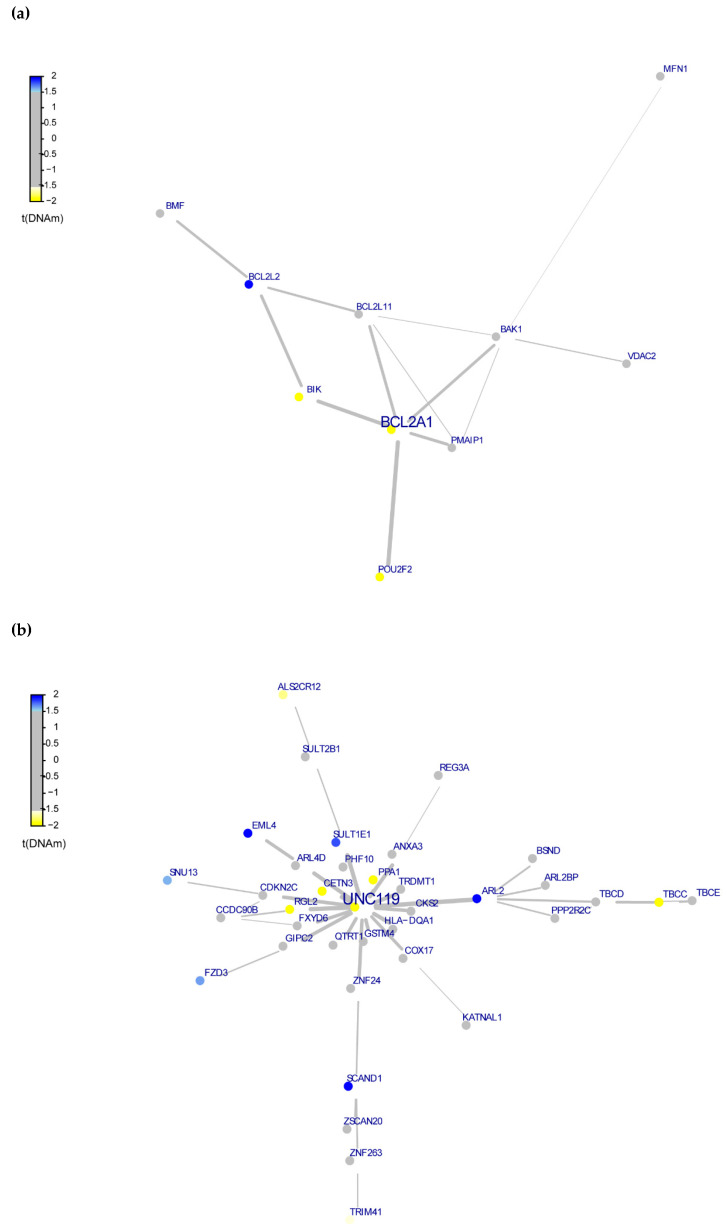

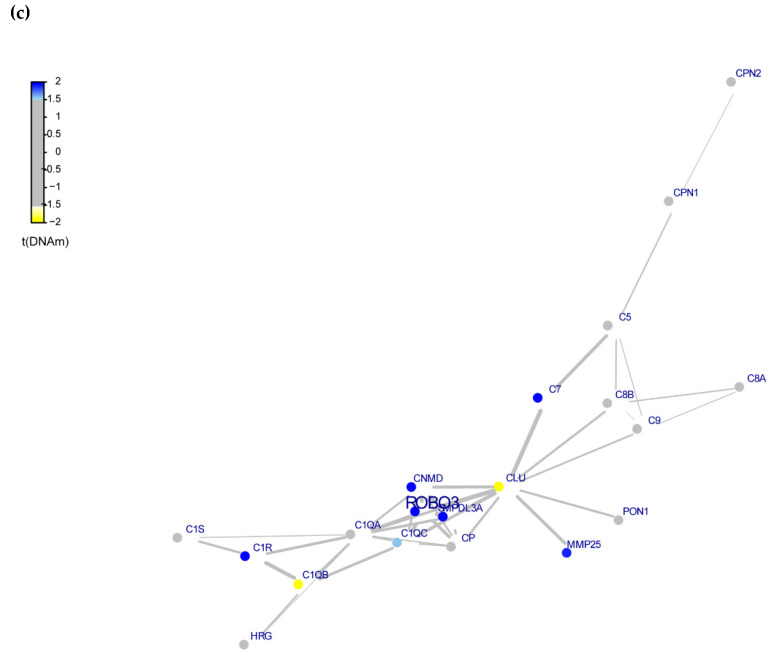
The functional epigenetic module of seed gene. (**a**) Module (BCL2A1), (**b**) Module (UNC19), and (**c**) ROBO3. Each dot is a gene and edges are the interaction in the PPI network, colors represent hypermethylation (blue) and hypomethylation (yellow) CpG sites in group B compared to group A samples. Gray dots are genes that showed no statistical significance in the comparison between groups or not represented in the initial analysis.

To investigate the pathways associated with DMPs, we performed the Functional Modules Analysis using the FEM package, which uses the properties of protein-protein interactions to build modules, then it performs gene set enrichment analysis for pathways within the modules. This resulted in 3 modules of functionally related genes (Table S4 and Figure 3), with two of these modules being enriched for genes from immune system pathways functions and stress response (Table S5).

**Figure 4:**
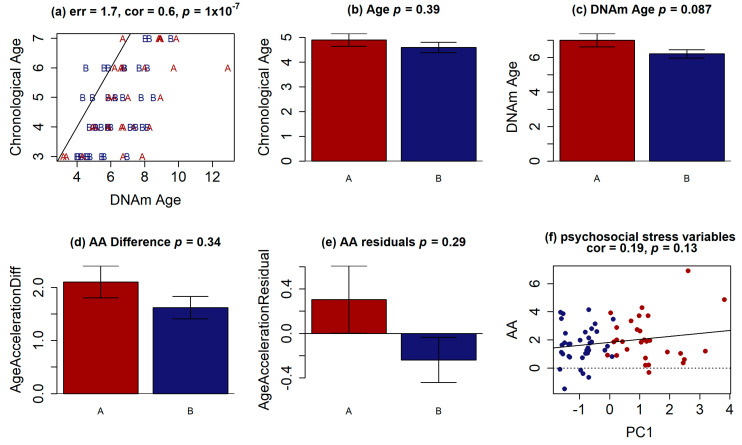
Age Acceleration analysis. (**a**) Scatter plot of chronological age (*y*-axis) and DNA methylation age (*x*-axis) each point represents a sample analyzed. Bar plot comparing (**b**) chronological age, (**c**) DNA methylation predicted age (**d**) Age acceleration difference (DNAm–Age) (**e**) Residual calculation from a linear model (DNAm~Age), the *p*-values represented at the top level in each graphics (Mann-Whitney test). (**f**) Scatter plot of age acceleration (*y*-axis) and PC1 (*x*-axis) which resumes the psychosocial stress each point represents a sample analyzed. In all graphics, group A is red and group B blue.

To investigate the postnatal stress burden, we used the epigenetic age, following Horvath’s method [36]. As we are using it as a proxy of postnatal stress exposure, we expected that individuals assign to different gradients of vulnerability according to our PC analysis will not have AA differences. We calculated the methylation age and observed that both groups presented higher epigenetic age than chronological age, without a statistically significant difference between the groups (*p* = 0.087) (Figure 4a–c). The same was observed for Age Acceleration (*p* = 0.342) (Figure 4d). Although not statistically significant, when comparing the values of PC1, which resumes the psychosocial stress variables, we found that it was (weakly) positively correlated to epigenetic age acceleration (cor = 0.19, *p* = 0.13, Figure 4f).

### 3.4. Whole-Exome Analysis

To investigate if individuals assign to different gradients of vulnerability according to our PC analysis are not a consequence of the presence of rare variants of higher impact, we performed a whole-exome analysis on a subset of children and their both parents (8 from A and 25 from B) that were already available in our laboratory. Of the 6625 selected deleterious variants (of which 1966 are on the 25% most intolerant RVIS list), 5512 are in patients in group B (RVIS: 1547, *de novo*: 18) and 2,578 in patients in group A (RVIS: 736, *de novo*: 8). Χ^2^-test found no difference between groups (*p*-value > 0.05).

### 3.5. Regression Analyses

Using phenotypes as dependent variables, we built linear regression models using sex, age acceleration, the two PV groups, and PC scores, to understand their predictive power regarding clinical severity and functionality since they have been associated with clinical severity and prognostic [19,28,79]. Clinical severity and functionality were estimated by Child Autism Ratio Scale (CARS) and Vineland scores, respectively. We also built models introducing the father’s age as covariable to represent a different source of the genetic contribution [80]. However, no significant model was associated with the phenotypes investigated.

First, we used the moderation analysis using the interaction between groups, and age acceleration. No significant model was observed using these parameters in the moderation analysis. However, using the variables without the moderation effect, the model was built to predict CARS based on age acceleration, groups, and sex, resulting in a significant regression equation (F(3, 62) = 2.889, *p*-value = 0.043), with an R^2^ = 0.12. In this model, only grouping was a significant predictor (Table 3). A small variation of severity can be explained by this model: being in group A decreased the CARS score to 3.79 on average.

Considering a dimensional approach, we performed moderation analysis with the PC scores instead of groups. Using the vulnerability scores, we built two models, one with only PC1 and PC2 and the other using the 3 PCs (PC1, PC2, and PC3) to have more than 70% of variability explained. The moderation analysis using the first model, sex, PC1, PC2, and age acceleration, resulted in a significant regression equation (F(8, 54) = 2.149, *p*-value = 0.047), with an adjusted R^2^ = 0.13 only considering the Vineland score as the outcome. The significant variables were AA:PC1 and AA:PC2 (Table 4). In the second model, applying the 3 first PCs, Vineland Total Score was significantly predicted (F(16, 46) = 2.761, *p*-value = 0.0036 and adjusted R^2^ = 0.31) mostly by PC3, Sex, AA:PC2, AA:PC3 and AA:PC1:PC3 variables (Table 4). In regards to this model using 3 PCs, moderation analysis using CARS as a dependent variable did not produce a significant model (F(16, 49) = 1.74, *p*-value = 0.071 and adjusted R^2^ = 0.15).

## 4. Discussion

In the present work, we showed how adverse environmental exposure during gestation and genetics could contribute to ASD vulnerability and including a biological measure as a proxy of adverse environmental exposure after birth we also presented their contribution to ASD clinical severity.

We searched among a clinically homogeneous group of ASD patients, principal components of ASD vulnerability, considering psychiatric family history representing a polygenic contribution and different gestation environmental exposure as entered features. Two final components explained 60% of the variability: one psychosocial (named PV, correlated with psychological stress factors and socioeconomic problems exposition) and the other biological (named BV, correlated with gestation problems and psychiatric family history). We arbitrarily divided the samples into two groups (A and B, above and below PV average respectively), but we cannot see a clear separation between groups, and the formed subgroups were mainly a consequence of the psychosocial exposure component reinforced by statistically differences when comparing Maternal Stress, Maternal Schooling and Social Class between groups. So, even individuals in group B having more familial history than in group A, which implies a larger polygenic genetic background [38,81], the subgroups were not represented by extremes of genetic background and environmental exposure as previously suggested [82]. It is important to note that out of 7 individuals with positive familial ASD history, 3 were in group B and 4 in group A, so we are not forming subgroups of simplex and complex ASD as already described in the literature [83]. Moreover, in the whole-exome analysis, we could not find differences in the burden of *de novo* and very rare variants with the higher impact between groups.

Genetic and environmental exposures were entered features used to achieve a dimension reduction so we expected that VMRs (variable methylated regions), polymorphic methylation sites that have been aggregated in genomic regions associated with Genetic, Environmental and GxE effects and independent of age would be enriched in any methylome comparison. Accordingly, differentially methylated positions between groups divided by PC1 were enriched by genetic VMRs or those representing the GxE interaction, that is, associated with SNPs (informative VMRs, and VMRs in EWAS studies) [20,23]. As we are looking at differentially methylated positions, we cannot affirm that one group has more G, E, or GxE VMRs than the other, but a different pattern provided by the CpG cluster analysis reinforces biological differences between groups. Moreover, there was an absence of DMP enrichment in rVarbase regulatory elements, but the presence of different elements in both groups suggests that these regions may be affected by common genomic variations in important regulatory regions in different ways. Additionally, DNA hypermethylation in bivalent domains was observed in group A with higher psychosocial stress vulnerability, epigenetic changes in bivalent domains under stress or environmental exposures were observed in cancer cells [84,85]. The differentially expressed DMPs were enriched in databases related to 2 different tissue VMRs (saliva and blood), and in Cells B, T, Fibroblasts, Glia, and Neuron VMRs, which means that these VMRs should not be associated with tissue-specific programming [23,25]. The fact that VMRs are age invariable and the enriched VMRs are not tissue-specific, is important to support their hole in the vulnerability components, considering that our methylation array has been performed when kids were 7 years of age. But it is important to note that enrichment in glial and neuron VMRs together with correspondence analysis between blood and brain suggest that the variation of these CpGs may also have a role in gene expression alterations in brain tissue [25].

In addition to our DMPs being enriched in genomic regions related to genetic and environmental effects, gene enrichment from immune system pathways functions and stress response, already associated with environmental exposition and ASD, were found when analyzing differently methylated gene promoters from epigenetic functional modules. During pregnancy complications as metabolic disorders like insulin resistance, obesity, and chronic hypertension are associated with systemic inflammation [86]. Obesity leads to a persistent state of low-grade inflammation through the recruitment and secretion of pro-inflammatory cytokines by hypertrophic adipocytes. Moreover, excessive body fat and hyperglycemia are sources of oxidative stress [87]. Psychosocial exposure also is associated with inflammation and oxidative stress [34]. Since the epigenetic clock was not calculated when individuals were born, it probably reflects stress accumulated throughout the whole life and not only during gestation. According, the AA was associated with PC1, but there is a lack of differences in the epigenetic clock between the groups. Methylation analysis showed that the DMPs were enriched in VMRs influenced by genetic or environmental factors that participate in pathways important to ASD, supporting our vulnerability components and suggesting that individuals with similar symptoms may have differences in their biological basis

To achieve our second goal, we used regression analysis considering CARS and Vineland measured at seven years old as outcomes and formed groups, vulnerability components, and AA as a proxy of adverse environmental factors after birth as predictors. In a categorical view, groups were associated with CARS at seven years of age, whereas AA and sex were not. In a dimensional view, PCs, AA, and sex showed interactions associated with Vineland. Evidence for this is clearer when we apply the regression analyses using vulnerability score as a numeric variable represented by PC1, PC2, and later PC3 loads. Using only the first two PCs as independent variables, Vineland Total Score could be associated with the vulnerability score moderated by AA, suggesting that vulnerability scores calculated through risk factors could explain in part the variation in Vineland Total Score. The property of vulnerability score as a continuum is more pronounced using the model with the three PCs loads. This model resulted in an association with Vineland Total Score with a larger adjusted R^2^, meaning that the variation of these variables can better explain the variation of Vineland Total Score.

This article has a relatively small sample of only Brazilian young patients with severe ASD and it may not reflect the total ASD population. A previous study showed that ASD with and without ID share underlying mechanisms, although they have unique etiologic components [88]. It is a transversal study done with data that relied on the quality of the information provided by the patients’ relatives. Part of the individuals had to be excluded due to missing information from the questionnaires. Another limitation is that the specific timing of stress exposure was not measured. Even considering these limitations, we showed how genetic and adverse gestation environmental factors could contribute to ASD vulnerability. Showed differences in methylation sites associated with inherited common genetic inheritance and environmental response to stress, as important pathways for ASD, corroborating our findings. Finally, the regression analysis confirmed that components of vulnerability including environmental influences after birth could contribute to explain phenotypic heterogeneity.

## 5. Conclusions

This paper suggests that individuals with similar symptoms and diagnoses may present differences in their pathophysiological basis and, the variability in phenotypes in the autism spectrum could be explained considering the vulnerability components and postnatal environmental exposure.

## Figures and Tables

**Table 1:** Principal Component Analysis of environmental factors.

Variables	PC 1	PC 2	PC 3	PC 4	PC 5
Variance (%)	35.1	25.3	18.2	12.5	8.9
Cumulative Variance (%)	35.1	60.4	78.6	91.1	100
Eigenvalue	1.76	1.26	0.91	0.62	0.44
Gestational Complications	−0.1	**0.78**	−0.52	0.25	−0.23
Maternal Stress	**0.7**	−0.07	−0.59	−0.16	0.36
Maternal Schooling	**0.74**	−0.1	0.24	0.62	0.02
Social Class	**0.82**	0.08	0.12	−0.36	−0.41
Psychiatric Family History	−0.17	**−0.8**	−0.47	0.15	−0.3

**Table 2:** Comparison of 11,879 DMPs with VMRs databases.

Database		Total	Overlap	*p*-Value	FE
Islam et al., 2019 [23]	**Different tissues**	**139,662**	**4549**	**<1 × 10^−4^**	**1.23**
	**Informative**	**8140**	**295**	**<1 × 10^−4^**	**1.59**
	meQTL	4980	143	0.16	1.09
Hachiya et al., 2017 [19]	**VMRs EWAS**	**269**	**17**	**<1 × 10^−4^**	**2.31**
Garg et al., 2018 [25]	**B-Cells**	**4367**	**214**	**<1 × 10^−4^**	**1.85**
	Environmental	804	27	0.12	1.28
	Fibroblasts	4788	149	0.05	1.15
	**Glia Cells**	**6990**	**221**	**2.1 × 10^−3^**	**1.21**
	**Neurons**	**7075**	**230**	**1 × 10^−4^**	**1.27**
	**T-Cells**	**8940**	**396**	**<1 × 10^−4^**	**1.72**

**Table 3:** Multiple regression models parameters.

	Coeff. Estimation	Sd. Error	*t*-Value	Pr (>|t|)
Intercept	35.2	2.1	16.92	<2 × 10^−16^ ***
AA	0.33	0.55	0.6	0.55
Group (0 = B, 1 = A)	−3.8	1.6	−2.38	0.02 *
Sex (0 = female, 1 = male)	3	2	1.48	0.14

* *p*-value ≤ 0.05, *** *p*-value ≤ 1 × 10^−3^.

**Table 4:** Summary of moderation analysis.

Model: Vineland~AA * PC1 * PC2 + Sex				
	Coeff. Estimation	Sd. Error	*t*-Value	Pr (>|t|)
Intercept	44.4	2.6	16.75	<2 × 10^−16^ ***
AA	1.25	0.84	1.49	0.14
PC1	2.2	1.2	1.82	0.07
PC2	0.7	1.7	0.43	0.67
Sex (0 = female, 1 = male)	4	2.7	1.50	0.14
AA:PC1	−1.04	0.43	−2.43	1.87 × 10^−2^ *
AA:PC2	−1.63	0.79	−2.05	4.49 × 10^−2^ *
PC1:PC2	−0.7	1.4	−0.52	0.6
AA:PC1:PC2	0.85	0.84	1.00	0.32
Model: Vineland~AA * PC1 * PC2 * PC3 + Sex				
Intercept	42.15	2.51	16.80	<2 × 10^−16^ ***
AA	0.69	0.84	0.82	0.42
PC1	1.11	1.11	1.00	0.32
PC2	2.32	1.73	1.34	0.19
PC3	−9.12	2.89	−3.16	2.82 × 10^−3^ **
Sex (0 = female, 1 = male)	7.71	2.70	2.85	6.46 × 10^−3^ **
AA:PC1	−0.47	0.43	−1.10	0.28
AA:PC2	−2.62	0.84	−3.11	3.19 × 10^−3^ **
PC1:PC2	−0.96	1.42	−0.68	0.50
AA:PC3	5.13	1.43	3.58	8.26 × 10^−4^ ***
PC1:PC3	3.39	1.95	1.74	0.09
PC2:PC3	−2.80	2.34	−1.20	0.24
AA:PC1:PC2	1.05	0.81	1.29	0.20
AA:PC1:PC3	−2.93	1.00	−2.93	5.25 × 10^−3^ **
AA:PC2:PC3	1.41	0.95	1.49	0.14
PC1:PC2:PC3	−0.97	2.16	−0.45	0.65

* *p*-value ≤ 0.05, ** *p*-value ≤ 0.01, *** *p*-value ≤ 1 × 10^−3^.

## Data Availability

Raw methylation database is available at the GEO database (GSE164563) and whole-exome sequenced reads are available at SRA PRJNA525890.

## Supplementary Materials

The following are available online at https://www.mdpi.com/article/10.3390/genes12091433/s1, Table S1: Score of environmental factors and familial genetic history, Table S2: Summary of descriptive statistics of Groups, Table S3: Type of rVarBase elements in DMPs, Table S4: Functional Epigenetic Modules, Table S5: Enrichment analysis of FEM Genes (GO, KEGG and Phenotype Database). Figure S1: Venn Diagram of correspondence analysis of DNA methylation between blood and brain.

## Appendix A

Social and economic factors: Maternal schooling [52] score was 1 for complete college education, 2 for incomplete college, 3 for complete high School, 4 for incomplete high school, 5 complete basic school, 6 incomplete basic school, and 7 for no education. Family social class [51,52] score was no points for mothers from A and B class, 1 point for from C and 2 points from D.

Maternal stress SCORE: Stress during gestation [14,54], familial fights, illness, or death in the family, verbal, or physical aggression, and emotional or depression symptoms during pregnancy [89] were evaluated. The presence of each represented 1 point in the final score that varied from 0 (no stress) to 6 (presence of all the above).

Toxic environmental exposures SCORE: Tobacco, alcohol and drug use, exposure to pesticides, and use of known deleterious medications during pregnancy—e.g., diazepam, valproic acid, fluoxetine, and sertraline [13,90,91]. The presence of each was worth 0 to 2 points, depending on frequency and quantity of exposure. The total sum represented a score from 0 to 10.

Familial Genetic risk SCORE: All psychiatric disorders reported from the proband families were combined in a single score [8,9]. The pathologies listed were: ASD, schizophrenia, depression, bipolar disorder, alcohol and/or drug abuse, obsessive-compulsive disorder, anxiety, attention deficit, and hyperactivity disorder. We used the combination of all the disorders to represent a polygenic risk of the psychiatric disorder [18]. Each score was balanced according to the degree of familial proximity to the proband. First-degree relatives had weight 3, second-degree 2, and third-degree 1. We also used reliability of information to weigh the score: if the family member was hospitalized for the psychiatric disorder (weight 3), if they used medications or did therapy because of the disorder (weight 2), and if none of the above (weight 1). The final score varied from 0 to 18. For Example Subject 18, (a) mother with depression with no use of medication and no treatment for the condition; (b) uncle with schizophrenia, hospitalized for the condition. The score was 9, calculated as follows: 3 (1st degree relative with depression) × 1 (low reliability) + 2 (2nd degree relative with schizophrenia) × 3 (high reliability).

Gestational complications SCORE: Eclampsia/preeclampsia, gestational diabetes, and excess weight gain during gestation (more than 12 kg) were used to form the score [86,87,90,92,93]. The presence of each was worth 1 point in the final score that varied from 0 to 3.

## Appendix B. VMRs Dataset Details

1. Islam et al. [23] with a systematic comparison of genome-wide DNAm patterns (DNAm variability, cross-tissue DNAm concordance, and genetic determinants of DNAm across two independent early life cohorts encompassing different ages) between matched pediatric buccal epithelial cells (BECs) and peripheral blood mononuclear cells (PBMCs). They overlapped CpGs that were identified as (a) informative (i.e., variable across individuals and correlated between BECs and PBMCs) (8140), (b) differentially methylated between matched tissues (139,662), or (c) under genetic influence (4980; i.e., number of unique CpGs associated with validated cismQTL across two cohorts;
2. Teh et al. [20], an examination of the relative influences of genotypic, environmental, and gene x environment interactive effects on the neonatal methylome. They studied the variation in genome-wide DNA methylation patterns in umbilical cord samples, genotyping, and measures of in utero environmental conditions and identified 1423 interindividual variably methylated regions (VMRs) across the 237 individuals. Methylation levels at 25% of the 1423 VMR-CpGs were best explained by genotype alone, while the rest were best explained by G × E models.
3. Garg et al. [25] performed a screen to identify regions of common epigenetic variation using population data derived from five different human cell types. They searched for clusters of probes with high inter-individual variability (VMRs) and explored the potential underlying factors associated with the regulation of VMRs using different strategies (VMRs influenced by genetic, environmental, and GxE effects), and
4. Hachiya et al. [19] catalog of EWAS VMRs.
