# Supplementary material for: Environmental Influences Measured by Epigenetic Clock and Vulnerability Components at Birth Impact Clinical ASD Heterogeneity
Source: Genes (Basel). 2021 Sep 17;12(9):1433. doi: 10.3390/genes12091433 (PMC8467464; doi:10.3390/genes12091433)
Supplement: Supplementary file 1 [file genes-12-01433-s001.zip › Figure S1.pdf]

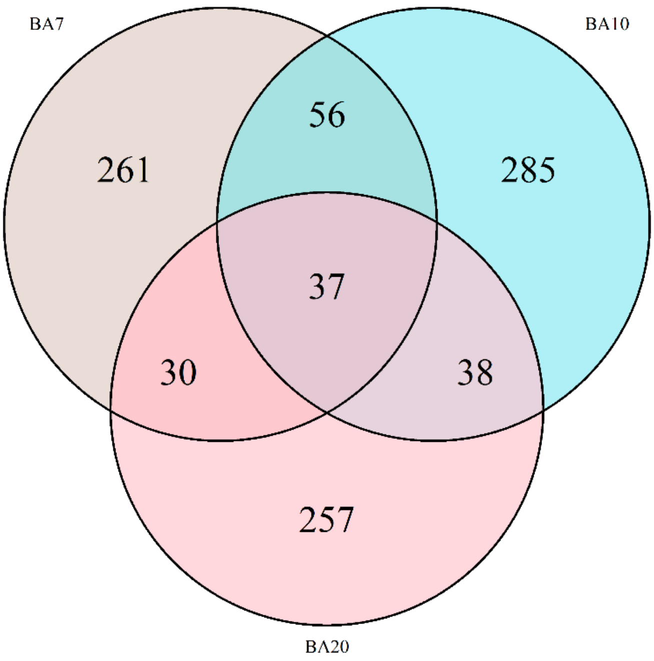

Figure S1. Venn Diagram of correspondence analysis of DNA methylation between blood and brain. Each circle represents a Broadman Area of the brain. The number of CpGs is defined as an overlapping of DMPs identified and children methylome and correlation $|r| \geq 0.5$ between blood and the respective brain area in BECon datasets.
