# Supplementary material for: Environmental Influences Measured by Epigenetic Clock and Vulnerability Components at Birth Impact Clinical ASD Heterogeneity
Source: Genes (Basel). 2021 Sep 17;12(9):1433. doi: 10.3390/genes12091433 (PMC8467464; doi:10.3390/genes12091433)
Supplement: Supplementary file 1 [file genes-12-01433-s001.zip › TableS1.pdf]

Table S1: Score of environmental factors and familial genetic history

ID	GestDisease	MatStress	MatSchool	SocialClass	GenetFH
ID01	1	0	2	0	10
ID02	1	3	6	0	12
ID04	1	2	4	1	6
ID05	0	2	3	0	9
ID07	0	1	3	0	8
ID08	1	0	3	0	0
ID09	0	0	3	0	6
ID10	0	5	3	1	9
ID11	0	1	6	0	0
ID12	1	0	3	0	0
ID14	3	2	3	1	0
ID15	1	0	3	0	0
ID16	1	0	3	0	0
ID17	1	0	3	0	8
ID19	1	0	1	0	0
ID20	1	1	6	2	3
ID21	0	1	1	0	12
ID22	1	3	4	2	3
ID23	0	3	1	1	18
ID24	0	0	3	2	6
ID25	0	0	3	1	0
ID26	1	0	3	1	4
ID27	0	0	3	0	0
ID28	1	0	3	0	10
ID29	2	0	1	1	0
ID30	0	0	3	0	0
ID31	0	0	3	2	1
ID32	0	0	3	0	11
ID33	0	0	3	1	6
ID34	0	3	6	2	9
ID35	1	1	4	0	6
ID36	0	0	1	0	6
ID37	1	0	1	0	6
ID38	1	0	1	0	4
ID39	1	1	3	0	16
ID42	0	1	3	2	6
ID43	1	0	1	1	12
ID46	0	0	2	0	12
ID47	0	1	5	1	13
ID48	3	1	1	0	6
ID49	0	4	3	2	0
ID50	1	0	1	0	5
ID51	0	0	1	0	7
ID52	1	2	4	1	0
ID53	2	0	3	0	0
ID54	1	0	3	1	0
ID56	1	0	1	0	2
ID57	1	0	1	0	0
ID58	0	3	1	0	9
ID59	0	1	1	0	12
ID60	0	3	3	1	6
ID61	2	4	3	0	6
ID62	1	0	5	0	10
ID63	1	2	1	0	3
ID64	2	3	3	1	0

ID65	2	1	3	1	0
ID66	1	2	1	0	0
ID68	2	3	3	1	0
ID69	0	4	6	2	0
ID71	0	0	3	1	0
ID72	2	0	2	0	6
ID73	0	0	3	0	2
ID74	0	0	3	1	0
ID75	1	0	1	0	4
ID77	0	2	3	1	0
ID78	2	4	3	2	2
ID80	1	1	3	2	3
