# Supplementary material for: Environmental Influences Measured by Epigenetic Clock and Vulnerability Components at Birth Impact Clinical ASD Heterogeneity
Source: Genes (Basel). 2021 Sep 17;12(9):1433. doi: 10.3390/genes12091433 (PMC8467464; doi:10.3390/genes12091433)
Supplement: Supplementary file 1 [file genes-12-01433-s001.zip › TableS2.pdf]

Table S2: Summary of descriptive statistics of Groups

PHENOTYPE	Group A			Group B			p-value**
	n	Mean	SD	n	Mean	SD	
Age	29	4.90	1.40	38	4.63	1.3	0.45
CARS	29	34.52	6.27	38	38.25	6.3	0.06
CBCL Internalizing Symptoms	29	12.03	4.75	38	10.37	5.3	0.12
CBCL Externalizing Symptoms	29	14.59	8.32	38	13.47	5.9	0.77
ADI	26	47.42	6.95	38	46.68	7.0	0.54
IQ	26	59.31	9.13	38	58.84	8.7	0.80
Vineland Total	26	48.85	7.96	38	49.97	9.3	0.95
Vineland Communication	27	44.81	7.30	37	48.70	9.4	0.26
Vineland Socialization	27	53.07	9.62	37	54.16	10.6	0.77
Vineland Daily Life Activities	27	51.41	13.07	37	49.38	12.9	0.92

*** Wilcoxon Mann-Whitney test was performed to Risk Scores and Phenotype measures*
