# Supplementary material for: Environmental Influences Measured by Epigenetic Clock and Vulnerability Components at Birth Impact Clinical ASD Heterogeneity
Source: Genes (Basel). 2021 Sep 17;12(9):1433. doi: 10.3390/genes12091433 (PMC8467464; doi:10.3390/genes12091433)
Supplement: Supplementary file 1 [file genes-12-01433-s001.zip › TableS3.pdf]

Table S3: Type of rVarBase elements in DMPs

Type*	group B	group A	Expected B (B vs A)	Expected A (B vs A)	p-value (B vs A)	Expected B (B vs Bck)	p-value (B vs bck)	Expected A (A vs Bck)
TssA	604	532	622	514	2.80E-01	552	2.10E-02	457
TssAFlnk	805	664	804	665	9.70E-01	735	**6.40E-03**	608
TxFlnk	455	189	352	292	**9.40E-17**	365	**1.10E-06**	299
Tx	533	338	477	394	**7.70E-05**	603	**2.80E-03**	498
TxWk	1281	936	1213	1004	**1.40E-03**	1321	2.20E-01	1092
EnhG	525	348	478	395	**9.50E-04**	553	2.20E-01	457
Enh	1085	918	1096	907	6.00E-01	1097	7.10E-01	908
ZNF/Rpts	194	140	183	151	2.30E-01	199	7.50E-01	164
Inactive region	18	8	14	12	2.00E-01	19	9.20E-01	16
TssBiv	326	368	380	314	**2.80E-05**	325	9.70E-01	270
BivFlnk	244	363	332	275	**2.10E-13**	247	8.50E-01	207
EnhBiv	379	532	499	412	**1.60E-16**	433	**7.00E-03**	361

*Classification performed by RoadMap Epigenomics

bck: background based on 450K Illumina, cl: Cluster

TssA: Active TSS; TssAFlnk: Flanking Active TSS; TxFlnk:Transcr. at gene 5' and 3'; Tx: Strong transcription; TxWk: W

p-value (A vs bck)
**2.40E-04**
1.70E-02
**4.70E-11**
**4.80E-14**
**1.20E-07**
**1.00E-07**
7.20E-01
5.80E-02
7.10E-02
**8.50E-10**
**1.00E-28**
**1.10E-20**

Veak
