# Supplementary material for: Environmental Influences Measured by Epigenetic Clock and Vulnerability Components at Birth Impact Clinical ASD Heterogeneity
Source: Genes (Basel). 2021 Sep 17;12(9):1433. doi: 10.3390/genes12091433 (PMC8467464; doi:10.3390/genes12091433)
Supplement: Supplementary file 1 [file genes-12-01433-s001.zip › TableS4.pdf]

Table S4: Functional Epigenetic Modules

Module (Seed Gene)	EntrezID	Gene Symbol	stat (DNAm)*	p-value
UNC119	9094	UNC119	-5.09	3.00E-06
	306	ANXA3	1.17	0.25
	379	ARL4D	1.50	0.14
	2948	GSTM4	0.79	0.43
	81890	QTRT1	-0.45	0.65
	7572	ZNF24	-0.65	0.52
	1031	CDKN2C	-0.79	0.43
	6783	SULT1E1	1.84	0.07
	5464	PPA1	-1.93	0.06
	1787	TRDMT1	-1.40	0.17
	10063	COX17	0.06	0.95
	1164	CKS2	-0.58	0.56
	5863	RGL2	-1.91	0.06
	53826	FXYD6	-0.50	0.62
	3117	HLA-DQA1	1.45	0.15
	55274	PHF10	0.06	0.95
	402	ARL2	2.27	0.03
	1070	CETN3	-2.00	0.05
	54810	GIPC2	0.67	0.50
	27436	EML4	3.88	0.00
	7809	BSND	0.79	0.43
	5522	PPP2R2C	-0.42	0.68
	23568	ARL2BP	-0.77	0.44
	4809	SNU13	1.62	0.11
	6820	SULT2B1	-0.36	0.72
	60492	CCDC90B	0.85	0.40
	84056	KATNAL1	1.27	0.21
	6904	TBCD	1.32	0.19
	6903	TBCC	-3.33	0.00
	6905	TBCE	-0.52	0.61
	7976	FZD3	1.66	0.10
	5068	REG3A	0.13	0.90
	51282	SCAND1	2.24	0.03
	7579	ZSCAN20	-1.11	0.27
	10127	ZNF263	0.52	0.60
	90933	TRIM41	-1.54	0.13
	130540	ALS2CR12	-1.69	0.10
BCL2A1	597	BCL2A1	-4.46	3.00E-05
	10018	BCL2L11	-0.69	0.49
	578	BAK1	-0.55	0.58
	5366	PMAIP1	-0.77	0.45
	638	BIK	-2	0.05
	5452	POU2F2	-2.7	0.01
	599	BCL2L2	3.34	1.00E-03
	90427	BMF	-1.06	0.29
	7417	VDAC2	-1.51	0.14
	55669	MFN1	0.09	0.93
ROBO3	64221	ROBO3	3.97	2.00E-04

11061	CNMD	2.01	0.05
1191	CLU	-2.8	0.01
712	C1QA	1.11	0.27
10924	SMPDL3A	3.01	4.00E-03
714	C1QC	1.57	0.12
1356	CP	-0.8	0.43
713	C1QB	-2.63	0.01
715	C1R	3.15	2.00E-03
64386	MMP25	1.92	0.06
716	C1S	-0.44	0.67
730	C7	3.65	5.00E-04
5444	PON1	0.75	0.45
3273	HRG	0.03	0.98
732	C8B	-0.64	0.52
727	C5	1.41	0.16
735	C9	-0.05	0.96
731	C8A	1.4	0.17
1369	CPN1	-0.8	0.43
1370	CPN2	-0.37	0.71

* stat (DNAm): estatística da metilação diferencia calculado pelo algoritimo FEM
