# Supplementary material for: Environmental Influences Measured by Epigenetic Clock and Vulnerability Components at Birth Impact Clinical ASD Heterogeneity
Source: Genes (Basel). 2021 Sep 17;12(9):1433. doi: 10.3390/genes12091433 (PMC8467464; doi:10.3390/genes12091433)
Supplement: Supplementary file 1 [file genes-12-01433-s001.zip › TableS5.pdf]

Table S5: Enrichment analysis of FEM Genes (GO, KEGG and Phenotype Database)

MOD	geneSet	size	overlap	expect	Ratio	pValue	FDR	Genes (EntrezGene ID)	Function	database
BCL2A1	GO:2001233	385	8	0.24	33.26	4.50E-12	**2.94E-09**	578, 597, 599, 638, 5366, 7417, 10018, 90427	regulation of apoptotic signaling pathway	GO: Biological Process
	GO:0097193	285	7	0.18	39.31	6.05E-11	**2.63E-08**	578, 597, 599, 5366, 7417, 10018, 90427	intrinsic apoptotic signaling pathway	GO: Biological Process
	GO:0008637	116	5	0.07	68.99	4.49E-09	**1.46E-06**	578, 638, 5366, 10018, 90427	apoptotic mitochondrial changes	GO: Biological Process
	GO:0007006	126	5	0.08	63.51	6.82E-09	**1.78E-06**	578, 5366, 10018, 55669, 90427	mitochondrial membrane organization	GO: Biological Process
	GO:0010821	176	5	0.11	45.47	3.66E-08	**7.96E-06**	578, 638, 5366, 10018, 90427	regulation of mitochondrion organization	GO: Biological Process
	GO:0038034	69	4	0.04	92.78	6.51E-08	**1.21E-05**	578, 597, 599, 10018	signal transduction in absence of ligand	GO: Biological Process
	GO:0097191	220	5	0.14	36.38	1.12E-07	**1.64E-05**	578, 597, 599, 5366, 10018	extrinsic apoptotic signaling pathway	GO: Biological Process
	GO:0090559	79	4	0.05	81.04	1.13E-07	**1.64E-05**	578, 5366, 10018, 90427	regulation of membrane permeability	GO: Biological Process
	GO:0043254	447	5	0.28	17.90	3.73E-06	**4.86E-04**	638, 5366, 7417, 10018, 90427	regulation of protein complex assembly	GO: Biological Process
	GO:0006839	237	4	0.15	27.01	9.18E-06	**1.09E-03**	578, 5366, 10018, 90427	mitochondrial transport	GO: Biological Process
	GO:0007548	266	4	0.17	24.07	1.45E-05	**1.57E-03**	578, 599, 638, 10018	sex differentiation	GO: Biological Process
	GO:0070585	137	3	0.09	35.05	7.05E-05	**7.07E-03**	5366, 10018, 90427	protein localization to mitochondrion	GO: Biological Process
	GO:0061458	428	4	0.27	14.96	9.32E-05	**8.68E-03**	578, 599, 638, 10018	reproductive system development	GO: Biological Process
	GO:1902742	31	2	0.02	103.26	1.62E-04	**1.32E-02**	578, 10018	apoptotic process involved in development	GO: Biological Process
	GO:0048872	245	3	0.15	19.60	3.93E-04	**3.01E-02**	578, 5366, 10018	homeostasis of number of cells	GO: Biological Process
	GO:0034976	268	3	0.17	17.92	5.11E-04	**3.70E-02**	578, 5366, 10018	response to endoplasmic reticulum stress	GO: Biological Process
	GO:0019867	206	8	0.13	62.16	2.89E-14	**3.76E-11**	578, 597, 599, 5366, 7417, 10018, 55669, 90427	outer membrane	GO: Cellular Component
	GO:0046982	468	4	0.29	13.68	1.32E-04	**1.15E-02**	578, 597, 599, 10018	protein heterodimerization activity	GO: Molecular Function
	hsa04215	33	3	0.04	75.44	6.48E-06	**1.83E-03**	10018, 5366, 578	Apoptosis	KEGG
	hsa04210	136	4	0.16	24.41	1.23E-05	**1.83E-03**	10018, 5366, 578, 597	Apoptosis	KEGG
	hsa05210	86	3	0.10	28.95	1.18E-04	**1.17E-02**	10018, 5366, 578	Colorectal cancer	KEGG
	hsa05206	299	4	0.36	11.10	2.70E-04	**2.01E-02**	10018, 578, 599, 90427	MicroRNAs in cancer	KEGG
	GO:0002697	381	13	0.48	27.31	0.00E+00	**0.00E+00**	712, 713, 714, 715, 716, 727, 730, 731, 732, 735, 1191, 1369, 1370	regulation of immune effector process	GO: Biological Process
	GO:0050727	361	13	0.45	28.82	0.00E+00	**0.00E+00**	712, 713, 714, 715, 716, 727, 730, 731, 732, 735, 1191, 1369, 1370	regulation of inflammatory response	GO: Biological Process
	GO:0051604	322	13	0.40	32.31	0.00E+00	**0.00E+00**	712, 713, 714, 715, 716, 727, 730, 731, 732, 735, 1191, 1369, 1370	protein maturation	GO: Biological Process

ROBO3	GO:0006959	242	14	0.30	46.30	0.00E+00	**0.00E+00**	712, 713, 714, 715, 716, 727, 730, 731, 732, 735, 1191, 1369, 1370, 3273	humoral immune response	GO: Biological Process
	GO:0002526	154	13	0.19	67.55	0.00E+00	**0.00E+00**	712, 713, 714, 715, 716, 727, 730, 731, 732, 735, 1191, 1369, 1370	acute inflammatory response	GO: Biological Process
	GO:0072376	92	13	0.11	113.08	0.00E+00	**0.00E+00**	712, 713, 714, 715, 716, 727, 730, 731, 732, 735, 1191, 1369, 1370	protein activation cascade	GO: Biological Process
	GO:0002449	238	11	0.30	36.99	8.88E-16	**1.45E-13**	712, 713, 714, 715, 716, 727, 730, 731, 732, 735, 1191	lymphocyte mediated immunity	GO: Biological Process
	GO:0002250	382	11	0.48	23.04	1.72E-13	**2.49E-11**	712, 713, 714, 715, 716, 727, 730, 731, 732, 735, 1191	adaptive immune response	GO: Biological Process
	GO:0019835	40	6	0.05	120.04	6.22E-12	**8.11E-10**	727, 730, 731, 732, 735, 3273	cytolysis	GO: Biological Process
	GO:0072562	108	11	0.13	81.51	0.00E+00	**0.00E+00**	713, 714, 715, 716, 731, 735, 1191, 1356, 1370, 3273, 5444	blood microparticle	GO: Cellular Component
	GO:0031012	496	7	0.62	11.29	1.44E-06	**1.70E-04**	712, 713, 714, 1191, 1370, 3273, 64386	extracellular matrix	GO: Cellular Component
	GO:0005581	87	3	0.11	27.59	1.65E-04	**1.54E-02**	712, 713, 714	collagen trimer	GO: Cellular Component
	GO:0017171	208	5	0.26	19.24	4.67E-06	**5.08E-04**	712, 713, 714, 715, 716	serine hydrolase activity	GO: Molecular Function
	GO:0004175	436	6	0.54	11.01	1.11E-05	**1.11E-03**	712, 713, 714, 715, 716, 64386	endopeptidase activity	GO: Molecular Function
	hsa04610	79	11	0.15	74.28	0.00E+00	**0.00E+00**	1191, 712, 713, 714, 715, 716, 727, 730, 731, 732, 735	Complement and coagulation cascades	KEGG
	hsa05020	35	8	0.07	121.94	2.22E-16	**3.30E-14**	712, 713, 714, 727, 730, 731, 732, 735	Prion diseases	KEGG
	hsa05322	133	10	0.25	40.11	2.22E-15	**2.20E-13**	712, 713, 714, 715, 716, 727, 730, 731, 732, 735	Systemic lupus erythematosus	KEGG
	hsa05150	56	6	0.10	57.16	3.87E-10	**2.87E-08**	712, 713, 714, 715, 716, 727	Staphylococcus aureus infection	KEGG
	hsa05133	76	6	0.14	42.12	2.56E-09	**1.52E-07**	712, 713, 714, 715, 716, 727	Pertussis	KEGG
	hsa05146	96	3	0.18	16.67	6.76E-04	**3.35E-02**	731, 732, 735	Amoebiasis	KEGG
	hsa05142	102	3	0.19	15.69	8.07E-04	**3.42E-02**	712, 713, 714	Chagas disease (American trypanosomiasis)	KEGG
	HP:0005339	23	10	0.09	113.77	0.00E+00	**0.00E+00**	712, 713, 714, 715, 716, 727, 730, 731, 732, 735	Abnormality of complement system	Human Phenotype Ontology
	HP:0004431	20	9	0.08	117.75	0.00E+00	**0.00E+00**	731, 732, 712, 713, 714, 715, 727, 730, 735	Complement deficiency	Human Phenotype Ontology

	HP:0005368	182	10	0.70	14.38	8.85E-11	**6.92E-08**	712, 713, 714, 715, 716, 727, 730, 731, 732, 735	Abnormality of humoral immunity	Human Phenotype Ontology
	HP:0002960	117	6	0.45	13.42	2.48E-06	**1.45E-03**	712, 713, 714, 715, 716, 731	Autoimmunity	Human Phenotype Ontology
	HP:0000123	33	4	0.13	31.72	5.30E-06	**2.49E-03**	712, 713, 714, 715	Nephritis	Human Phenotype Ontology
	GO:0031109	108	3	0.23	13.08	1.54E-03	3.34E-01	402, 1070, 6904	microtubule polymerization or depolymerization	GO: Biological Process
	GO:0006399	183	3	0.39	7.72	6.78E-03	1.00E+00	1787, 5464, 81890	tRNA metabolic process	GO: Biological Process
	GO:0006457	210	3	0.45	6.72	9.88E-03	1.00E+00	6903, 6904, 6905	protein folding	GO: Biological Process
	GO:0016328	56	3	0.12	25.22	2.25E-04	2.93E-01	402, 6904, 7976	lateral plasma membrane	GO: Cellular Component
	GO:0031970	87	3	0.18	16.23	8.22E-04	3.34E-01	402, 10063, 23568	organelle envelope lumen	GO: Cellular Component
	GO:0005874	402	5	0.85	5.85	1.49E-03	3.34E-01	6903, 6904, 6905, 27436, 84056	microtubule	GO: Cellular Component
	GO:0045171	59	2	0.13	15.96	6.95E-03	1.00E+00	2948, 9094	intercellular bridge	GO: Cellular Component
	GO:0015631	321	5	0.68	7.33	5.43E-04	3.34E-01	1070, 6903, 6904, 27436, 84056	tubulin binding	GO: Molecular Function
	GO:0051087	100	3	0.21	14.12	1.23E-03	3.34E-01	6903, 6904, 6905	chaperone binding	GO: Molecular Function
	GO:0016782	70	2	0.15	13.45	9.66E-03	1.00E+00	6783, 6820	transferase activity, transferring sulfur-containing groups	GO: Molecular Function
	hsa05225	168	3	0.31	9.53	3.39E-03	8.06E-01	2948, 55274, 7976	Hepatocellular carcinoma	KEGG
	hsa00140	60	2	0.11	17.78	5.43E-03	8.06E-01	6783, 6820	Steroid hormone biosynthesis	KEGG
	hsa05166	255	3	0.48	6.28	1.08E-02	1.00E+00	1031, 3117, 7976	Human T-cell leukemia virus 1 infection	KEGG
	hsa00190	133	2	0.25	8.02	2.49E-02	1.00E+00	10063, 5464	Oxidative phosphorylation	KEGG
	hsa04390	154	2	0.29	6.93	3.27E-02	1.00E+00	5522, 7976	Hippo signaling pathway	KEGG
UNC119	hsa04934	154	2	0.29	6.93	3.27E-02	1.00E+00	1031, 7976	Cushing syndrome	KEGG
	hsa05310	31	1	0.06	17.21	5.66E-02	1.00E+00	3117	Asthma	KEGG
	hsa05330	38	1	0.07	14.04	6.90E-02	1.00E+00	3117	Allograft rejection	KEGG
	hsa05332	41	1	0.08	13.01	7.42E-02	1.00E+00	3117	Graft-versus-host disease	KEGG
	hsa04940	43	1	0.08	12.41	7.77E-02	1.00E+00	3117	Type I diabetes mellitus	KEGG
	HP:0002717	33	2	0.07	29.73	1.86E-03	1.00E+00	7809, 1031	Adrenal overactivity	Human Phenotype Ontology

HP:0006829	60	2	0.12	16.35	6.07E-03	1.00E+00	6904, 6905	Severe muscular hypotonia	Human Phenotype Ontology
HP:0011767	60	2	0.12	16.35	6.07E-03	1.00E+00	1031, 6905	Abnormality of the parathyroid physiology	Human Phenotype Ontology
HP:0002510	62	2	0.13	15.83	6.47E-03	1.00E+00	6904, 6905	Spastic tetraplegia	Human Phenotype Ontology
HP:0000828	67	2	0.14	14.65	7.52E-03	1.00E+00	1031, 6905	Abnormality of the parathyroid gland	Human Phenotype Ontology
HP:0010929	217	3	0.44	6.78	7.59E-03	1.00E+00	1031, 6905, 7809	Abnormality of cation homeostasis	Human Phenotype Ontology
HP:0003111	241	3	0.49	6.11	1.02E-02	1.00E+00	1031, 6905, 7809	Abnormality of ion homeostasis	Human Phenotype Ontology
HP:0040077	91	2	0.19	10.78	1.36E-02	1.00E+00	1031, 6905	Abnormal concentration of calcium in blood	Human Phenotype Ontology
HP:0010783	101	2	0.21	9.72	1.66E-02	1.00E+00	1031, 6820	Erythema	Human Phenotype Ontology
HP:0003117	316	3	0.64	4.66	2.13E-02	1.00E+00	1031, 23568, 6905	Abnormality of circulating hormone level	Human Phenotype Ontology
